# Circulating microRNAs and Their Role in Multiple Myeloma

**DOI:** 10.3390/ncrna5020037

**Published:** 2019-05-02

**Authors:** Cinzia Federico, Antonio Sacco, Angelo Belotti, Rossella Ribolla, Valeria Cancelli, Arianna Giacomini, Roberto Ronca, Marco Chiarini, Luisa Imberti, Mirella Marini, Giuseppe Rossi, Marco Presta, Bruno Paiva, Aldo M. Roccaro

**Affiliations:** 1 Clinical Research Development and Phase I Unit, ASST Spedali Civili di Brescia, 25123 Brescia, Italy; cinziafederico84@gmail.com (C.F.); saccoantonio74@gmail.com (A.S.); 2 CREA Laboratory, ASST Spedali Civili di Brescia, 25123 Brescia, Italy; mrchiarini@gmail.com (M.C.); luisa.imberti@asst-spedalicivili.it (L.I.); mirella.marini@asst-spedalicivili.it (M.M.); giuseppe.rossi@asst-spedalicivili.it (G.R.); 3 Department of Hematology, ASST Spedali Civili di Brescia, 25123 Brescia, Italy; ange.belotti@gmail.com (A.B.); rossella.ribolla@hotmail.it (R.R.); valeriacancelli@me.com (V.C.); 4 Department of Molecular and Translational Medicine, University of Brescia Medical School, 25123 Brescia, Italy; arianna.giacomini@unibs.it (A.G.); roberto.ronca@unibs.it (R.R.); marco.presta@unibs.it (M.P.); 5 Clinical Chemistry Laboratory, Department of Diagnostics, ASST Spedali Civili di Brescia, 25123 Brescia, Italy; 6 Laboratory for Stem Cells Manipulation and Cryopreservation, Department of Transfusion Medicine, ASST Spedali Civili di Brescia, 25123 Brescia, Italy; 7 Clinical and Translational Medicine, Clínica Universidad de Navarra, 31008 Pamplona, Spain; bpaiva@unav.es

**Keywords:** multiple myeloma, plasma Cell dyscrasia, miRNAs, circulating miRNAs, circulating exosomal-miRNAs

## Abstract

Multiple myeloma (MM) is a plasma cell dyscrasia characterized by bone marrow infiltration of clonal plasma cells. The recent literature has clearly demonstrated clonal heterogeneity in terms of both the genomic and transcriptomic signature of the tumor. Of note, novel studies have also highlighted the importance of the functional cross-talk between the tumor clone and the surrounding bone marrow milieu, as a relevant player of MM pathogenesis. These findings have certainly enhanced our understanding of the underlying mechanisms supporting MM pathogenesis and disease progression. Within the specific field of small non-coding RNA-research, recent studies have provided evidence for considering microRNAs as a crucial regulator of MM biology and, in this context, circulating microRNAs have been shown to potentially contribute to prognostic stratification of MM patients. The present review will summarize the most recent studies within the specific topic of microRNAs and circulating microRNAs in MM.

## 1. Introduction

Multiple myeloma (MM) represents the second most common hematological malignancy [1]. It is a B-cell disorder characterized by clonal growth and accumulation of malignant plasma cells (PCs) within the bone marrow (BM), and the presence of a monoclonal immunoglobulin in the serum and/or urine [2,3,4]. An active disease stage presents with renal failure, anemia, hypercalcemia, and bone lesions. It is thought that myeloma cells initially create an isolated plasmacytoma and then disseminate and engraft within distant sites throughout the skeleton, leading to multiple bone lytic lesions. This process indicates the ability of clonal PCs to disseminate from one site of the BM to another, going through the peripheral blood [2,3,4]. Moreover, the growth of clonal cells within the marrow: (i) Disrupts the equilibrium between bone-forming osteoblasts’ and bone-resorbing osteoclasts’ activity, which is crucial in the metabolism and turnover of bone tissues; and (ii) triggers inflammatory cytokines, such as interleukin-3 (IL-3), interleukin-6 (IL-6), and macrophage inflammatory proteins-1α (MIP-1α) as well as the receptor activator of nuclear factor-kB ligand (RANK-L), thus promoting osteoclast activation [5,6].

MM is commonly preceded by the asymptomatic pre-malignant monoclonal gammopathy of undetermined significance (MGUS), defined by the presence of serum M protein < 30 g/dL, clonal BM PCs infiltration < 10%, with no development of renal insufficiency, anemia, bone lytic lesions, hypercalcemia, or amyloidosis [2]. For unclear reasons, MGUS can remain stable for years and no treatment is required, or can evolve to MM or other related hematological disorders. Moreover, MM may also progress to plasma cell leukemia (PCL) and extramedullary myeloma, which are BM-independent conditions presenting a substantial increase of PCs in the blood with plasmacytomas outside the BM [7]. The intermediate stage between MGUS and MM is the smoldering MM (SMM), characterized by a 10% to 60% presence of clonal BM PCs, and serum or urinary M protein with levels ≥30 g/dL and ≥500 mg, respectively. At this stage, patients do not present with myeloma-defining events or amyloidosis [7,8]. The risk of progression from MM to SMM is around 10% per year for the first five years, 5% per year during the subsequent five, and 1% per year for the following 10 years thereafter [9,10]. 

MM onset and development is driven by several genetic abnormalities, gains or losses of chromosomes, as well as point mutations [7]. MM patients may display either cytogenetic alterations, such as a 17p(p53) deletion or translocation t(4;14), or gene mutations leading to the harmful activation of a plethora of cellular signaling pathways. The most frequently mutated genes in MM patients are: *KRAS* (23%), *NRAS* (20%), *FAM46C* (11%), *DIS3* (11%), and *TP53* (8%); and less frequent, but recurrent mutated genes are: *BRAF*, *TRAF3*, *PRDM1*, *CYLD*, *RB1*, *IRF4*, *EGR1*, *MAX*, *HIST1H1E*, and *ACTG1* [11,12]. In addition, epigenetic changes, including DNA methylation, chromatin structure, and miRNA deregulation, have been shown to be involved in MM evolution [2]. Moreover, the BM microenvironment actively participates in the differentiation, migration, proliferation, survival, and drug-resistance of the MM cells [13]. The BM niche is a heterogeneous environment that includes hematopoietic and non-hematopoietic cells (e.g., fibroblasts, endothelial, inflammatory, immune, and BM stomal cells, osteoclasts, and osteoblasts). Within the BM, the non-cellular compartment is composed of the extracellular matrix and the liquid milieu, including cytokines, growth factors, and chemokines [13,14]. All these components are non-malignant per se and constitute a connective and supportive tissue. The mutual interaction of BM components with cancer cells may alter the behavior of the entire environment. Specifically, the homing and the continuous trafficking in and out of the BM by the malignant PCs triggers a pro-survival loop responsible for the progression of the disease [15]. Despite the introduction of molecular-targeted therapies (such as proteasome and histone deacetylase inhibitors, immunomodulatory drugs, and monoclonal antibodies) that have joined the traditional therapies (alkylating agents, anthracyclines, and corticosteroids) along with autologous haemopoietic stem cell transplantation, improving the response rate and overall survival of patients, MM remains incurable [2,16]. Most of the patients relapse, becoming refractory to treatments due to drug resistance or development of minimal-residual disease [17]. 

Accepted diagnostic criteria to detect MGUS, SMM, and MM are based on: (i) The detection of M protein levels in serum and/or urine, (ii) the assessment of the BM infiltration of clonal PCs, and (iii) the validation of myeloma-defining events, including biomarker assessment and CRAB features (hypercalcemia, renal dysfunction, anemia, bone destruction) [4]. Moreover, several prognostic markers have been proposed so far to predict clinical outcome and to stratify patients in distinct risk classes [17]. The assessment of albumin/β2-microglobulin protein levels in the peripheral blood at the time of diagnosis have led to the development of the International Staging System (ISS). The ISS score segregates patients into three different groups: (i) ISS stage I group includes patients with albumin/β2-microglobulin protein levels ≥ 3.5 g/dL and <3.5 mg/L, respectively (median overall survival of 62 months); (ii) ISS stage II group includes patients not considered in stage I or III (median survival of 44 months); and (iii) ISS stage III group includes patients with β2-microglobulin protein level ≥ 5.5 mg/L (median survival of 29 months) [18]. Furthermore, the identification of chromosomal abnormalities (CA) has been adopted as a further prognostic stratification biomarker for newly-diagnosed MM patients. In particular, interphase fluorescence in situ hybridization (iFISH) revealed that the presence of deletion 17p [del(17p)] or translocation t(4;14), and t(14;16) was associated with a median overall survival of 24.5 months compared to a median overall survival of 50.5 months for patients lacking genetic alterations [19]. Another biomarker in MM is the serum lactate dehydrogenase (LDH), of which levels above the upper limit of normal indicate an increased disease aggressiveness [20]. In addition, other prognostic factors associated with very-high-risk MM have been identified, such as age, the presence of plasma cell leukemia, and high plasma cell labeling index [21,22,23]. Recently, the revised ISS (R-ISS) combined the ISS risk stratification algorithm with CA and LDH data, aiming to improve the stratification of patients, by defining subgroups of patients with different prognoses [24].

Even though these advances in the prognostic options have been accomplished, patients’ responses and outcomes remain highly heterogeneous. Additional biomarkers are needed to further stratify patients and improve response to therapy.

## 2. Overview of miRNA Processing

MicroRNAs (miRNAs) have gained attention within the field of MM research due to their pivotal role in the regulation of several cellular processes implicated in plasma cell development and in myelomagenesis [25]. MiRNAs are short non-coding RNAs (19–25 nucleotides) tuning gene expression at the post-transcriptional level, by binding the 3′-untranslated regions (3′-UTRs) of target RNAs (mRNAs) [26]. MiRNAs biogenesis starts in the nucleus where they are transcribed by RNA polymerase II into pri-miRNAs, containing a cap structure at the 5′-end and are poly-adenylated at the 3′-end [27]. The pri-miRNAs also contain specific hairpin-shaped stem-loop structures of ~70 nucleotides, which are recognized and cleaved by a nuclear microprocessor complex, consisting of two RNase III endonucleases, Drosha and Dicer, and the essential DiGeorge syndrome critical region gene 8 binding protein (DGCR8/Pasha) [28,29]. The resulting pre-miRNAs have a hairpin structure of about ~70 nucleotides, and is transported to the cytoplasm by Exportin-5 and its co-factor, RanGTP [30]. Here, they are converted into short, imperfect, double-stranded miRNAs duplexes of 19 to 24 nucleotides long (miRNA:miRNA*) by the RNase III endonuclease, Dicer-1, and its cofactor, transactivating the response RNA binding protein (TRBP) [31]. Thereafter, the miRNA duplex is unwound and the mature miRNA strand, along with the Argonaute (Ago2) protein, form a complex with the RNA-induced silencing complex (RISC), while miRNA* is degraded. The single stranded miRNA binds to the 3′-UTR of target mRNAs and depending on the degree of complementarity, miRNA binding to 3′-UTR represses translation or induces deadenylation and mRNA decay [32,33]. Moreover, they are crucial in a variety of cell functions, including development, differentiation, proliferation, metabolism, apoptosis, and senescence [34,35].

## 3. Role of miRNAs in MM

miRNAs may act as crucial regulators of cancer onset and progression by behaving as oncogenic modulators or tumor suppressors: miRNA deregulation has been linked to the pathogenesis of several malignancies, including MM [36,37]. Tumor suppressor miRNAs are generally down-regulated and/or deleted in tumor cells and their targets are mRNAs actively involved in proliferation and survival mechanisms of tumor cells. Conversely, oncogenic miRNAs are over-expressed in tumor cells and promote tumor development by targeting tumor suppressor genes [38]. Consequently, the replacement of down-regulated miRNAs or the inhibition of over-expressed miRNAs could represent the rationale for an miRNA-based therapy in cancer [34,36].

Several research groups have investigated the expression profile of miRNAs both in vitro and in vivo by using MM cell lines or CD138 positive bone marrow plasma cells derived from MM patients or healthy controls, in order to provide insights into the functional role of miRNAs in MM. Of note, miR-21, miR-17-92, miR-106b-25 cluster, miR-32, and miR-181a/b were found up-regulated in MM cell lines and primary tumors compared to normal PCs. Moreover, miR-21, members of the miR-106b-25 cluster, and miR-181a/b were also up-regulated in MGUS patients. Additionally, by comparing MGUS and MM samples with normal PCs, only the miR-32 and miR-17-92 cluster were up-regulated in MM samples and MM cell lines as compared to normal PCs or MGUS, thus suggesting their plausible involvement in malignant transformation of MGUS to MM [39]. A comprehensive investigation on the role of miRNAs in MM has also been provided by performing expression profiling analysis of primary CD138+ cells from patients with relapsed/refractory MM and their normal counterparts, describing an overexpression of miR-222, miR-221, miR-382, miR181a, and miR-181b, together with lower expression of miR-15a and miR-16-1. miRNA-15a and -16-1 are located on chromosome 13q14, an area commonly deleted in MM. Indeed, a total absence of miRNA-15a and -16-1 was found in those patients with chromosome 13 deletion, while their levels were significantly decreased in the remaining patients without del(13) [40].

Moreover, functional studies showed that the replacement of synthetic pre-miR-15a and -16 in MM cells resulted in inhibition of pro-survival factors, such as AKT, MAPK, ribosomal-protein-S6, and NF-kB, leading to reduced MM cell proliferation and growth shown both in vitro and in vivo. Moreover, enforced expression of miR-15a and -16 also inhibited VEGF secretion, thus inhibiting MM cell-dependent endothelial cell growth and capillary formation, reduced migration and adhesion of MM cells to the BM milieu in vitro, and inhibited MM tumor progression in vivo [40,41]. Further studies have provided evidence of miRNAs deregulation and their involvement in MM pathogenesis. Among deregulated miRNAs in MM, the role of miR-29b is relevant. It has been demonstrated that restoring miR-29b expression in MM cells induced anti-proliferative and pro-apoptotic effects, either by targeting mRNAs of CDK6 and MCL-1, usually overexpressed in MM, or mRNA of Sp1, a transcription factor with oncogenic activity in MM [42] and Waldenstrom’s macroglobulinemia [43]. Constitutive expression of miR-29b also down-modulated mRNAs coding for HDAC4, DNMT-3A, and DNMT-3B, thus highlighting its epigenetic activity [44,45]. Consistent with its DNMT-inhibitory activity, miR-29b mimics induced CpG promoter demethylation of tumor suppressor, SOCS1 [46]. Moreover, the same research group demonstrated that aberrant deacetylation of miR-29a/b-1 promoter induced by HDACs, or aberrant miR-29a/b-1 promoter trimethylation at Lysine 27 of histone H3 by EZH2 mediates the silencing of miR-29b in myeloma [47]. A progressive decrease in miR-29b levels was also observed during human osteoclast differentiation in vitro, thus suggesting its involvement in bone metabolism. MM-associated bone disease is generally characterized by increased osteoclast activity and suppressed osteoblast function. Interestingly, miR-29b ectopic expression strongly impaired human osteoclasts’ differentiation, overcoming their activation induced by MM cells. Indeed, miR29b restoration downregulates NAFTc-1, abrogates the expression of TRAcP, CTSK, and MMP9, as well as actin ring rearrangement and their resorbing activity, through the targeting of MMP2 and c-FOS mRNAs [48]. Another anti-MM miRNA is miR-125b-5p, which was shown to exert tumor-suppressing activity in vitro and in vivo via direct targeting of the oncogenic IRF4 and its downstream effector, BLIMP-1. Moreover, the enforced expression of miR-125b-5p significantly decreases caspase 10, cFLIP, and c-Myc levels [49]. Of note, Myc is an important factor in MM, which was recently identified as deregulated in up to 49% of MM patients, including newly-diagnosed and previously-treated patients [2]. miR-23b is down-regulated in MM and behaves as a tumor suppressor. Restoration of miR-23b levels significantly reduced MM cell proliferation and survival, by inducing caspase-3/7 activity over time and abrogating the expression of Sp1-driven NF-kB. Interestingly, it has also been demonstrated that miR-23b transcription is under c-Myc control and the c-Myc/miR-23b/Sp1 feed-forward loop is critical in myeloma growth and survival [50]. 

On the contrary, the first oncogenic miRNA described in MM was miR-21. It acts as a pro-survival miRNA in myeloma and its expression levels are controlled by STAT3-IL6 [51]. It has been shown that miR-21 inhibition induced apoptosis and reduced tumor cell growth through overexpression of PTEN, Rho-B, and BTG2 [52]. Moreover, miR-21 inhibition restored the RANK-L/osteoprotegerin ratio in BM stromal cells co-cultured with MM cells, by decreasing RANK-L production and by increasing osteoprotegerin production, thus resulting in decreased osteoclast resorption [53]. As mentioned before, the miR-106/25 cluster was found up-regulated in MGUS patients compared to healthy subjects. The miR-106b/25 is a cluster encoding for miR-106b, miR-93, and miR-25 mature miRNAs and Gu et al. showed that seed-targeting tiny anti-miR-106b/25 decreased MM cell viability by reducing the expression of the target involved in the signaling pathways regulating proliferation, such as MAPK [54]. The role of the miR-17-92 cluster (also called OncomiR-1) is pivotal in MM, which was found significantly overexpressed in MM patients [39]. Several studies positively correlated miR-17-92 expression to MYC up-regulation during myeloma evolution [39,55], highlighting its tumor-promoting role. This has been linked to either the targeting of BIM and JAK/STAT-related genes (SOCS1, SOCS3, and SOCS5) as well as the modulation of IL-6 transduction [39,51,56]. It has been recently designed and developed a locked nucleic acid gapmeR antisense oligonucleotides (MIR17PTi) able to induce ribonuclease H-mediated degradation of MIR17HG primary transcripts and to prevent the biogenesis of all mature miRNAs of the miR-17-92 cluster (miR-17/-18a/-19a/-20a/-19b1/-92a1). Interestingly, MIR17PTi significantly impaired (i) the pro-survival Myc/miR-17-92 feed-forward loops in patient-derived MM cells, (ii) induced Myc-dependent synthetic lethality, and (iii) inhibited MM growth in both NOD SCID mice bearing subcutaneous MM xenografts and in a SCID-*hu* model [57]. Among oncogenic miRNAs, miR-125a-5p was found specifically up-regulated in t(4;14) patients, and its inhibition reduced cell proliferation and induced apoptosis, counteracting the tumor promoting effect of BM stromal cells [58]. Of note, members of the miR-99b/let-7e/miR-125a cluster, or of its paralog, also up-regulated in t(4;14) patients, were connected with the specific transcription factors, PBX1 and CEBPA, and several target genes potentially implicated in myeloma pathogenesis [59]. A list of the most representative deregulated miRNAs in MM cells is reported in Table 1.

Recently, transcripts longer than 200 nucleotides, also designated as long non-coding RNAs, have emerged as regulators of gene expression and have been implicated in MM [37,60]. The first identified and extensively characterized lncRNA is MALAT1, which has been found overexpressed in solid and hematologic malignancies [61]. Notably, MALAT1 silencing in MM cells through LNA gapmeR antisense olignoculeotides resulted in up-regulation of the tumor suppressor, miR-29b [47], and significantly inhibits tumor growth both in vitro and in vivo by proteasome blockade [62].

## 4. Circulating miRNAs

The identification of a wide array of miRNAs in various body fluids, such as plasma, serum, saliva, and urine [63], has grabbed the attention of the scientific community for its use as a minimally invasive prognostic or predictive marker for MM. Indeed, they possess several features that could facilitate their translation into clinical usage. Particularly, circulating miRNAs are not susceptible to enzymatic degradation due to their stable association with RNA-binding proteins (Argonaute2, nucleophosmin-1) [64,65], or with high-and low-density lipoproteins (HDLs and LDLs) [66], or because they are encapsulated in extracellular vesicles, such as exosomes, apoptotic bodies, and shedding vesicles [67]. Moreover, they are easily quantifiable in clinical samples and their stability was demonstrated at different pH levels or temperature (boiling, freeze-thawing) conditions, and long-term storage [68]. As aforementioned, miRNAs per se possess biological functions and their deregulation has been linked with tumorigenesis [38,69]. During the last years, several studies have investigated the potential role of circulating miRNAs as predictive, prognostic, and diagnostic biomarkers in MM [70]. Indeed, they could represent a less-invasive approach compared to BM-derived PCs from human biopsies, by helping the further stratification of MM patients.

In 2012, the first study on circulating miRNAs revealed a pattern of six miRNAs (miR-451, miR-638, miR-720, miR-1246, miR-1308, miR-1915) differentially expressed in MGUS, MM patients, and healthy controls. Particularly, the combination of miR-720 and miR-1308 was consistent in distinguishing MGUS and MM patients from normal counterparts, while the combination of miR-1246 and miR-1308 distinguished MGUS and MM patients [71].

In the same year, different research groups investigated the utility of serum miRNAs as prognostic tools. The authors identified miR-92a as a MM biomarker able to discriminate healthy controls from MM patients. Among symptomatic MM patients, miR-92a was found significantly down-regulated with respect to the stage of the disease and their response to therapy. Indeed, no difference in plasma miR-92a levels between MGUS and SMM patients was found, while the plasma miR-92a levels were significantly different between patients with SMM and symptomatic myeloma, suggesting that the level of plasma miR-92a could reflect the pathological condition of patients and could be helpful for deciding when to initiate chemotherapy [72]. Another study showed higher levels of six miRNAs (miR-148a, miR-181a, miR-20a, miR-221, miR-625, miR-99b) in the peripheral blood of newly diagnosed MM patients compared with healthy donors. Among these, the expression levels of miR-99b and miR-221 were associated with a t(4; 14) translocation and (13q) deletion, respectively. Moreover, higher levels of miR-20a and miR-148a were linked with a shorter relapse-free survival, thus suggesting their prognostic value [73]. Other studies reported on the up-regulation of miR-142-5p, miR-660, and miR-29a in the serum of MM patients compared to healthy controls, identifying miR-29 as a biomarker for MM patients with a sensitivity of 88% and a specificity of 70% from healthy controls [74]. The same research group detected five deregulated serum miRNAs (miR-744, miR130a, miR-34a, let-7d, and let-7e) differently expressed in MGUS, newly diagnosed, and relapsed MM patients compared to healthy donors. Particularly, the combination of miR-34a and let-7e differentiated MM from healthy donors with a sensitivity of 80.6% and a specificity of 86.7% and MGUS from healthy donors with a sensitivity of 91.1% and a specificity of 96.7%. They also showed that lower levels of let-7e along with miR-744 correlated with shorter survival and worse time to progression of MM patients [75]. miR-92a, -30a, and -451 have been reported to be down-regulated while miR-720 was shown to be up-regulated in MM patients compared with healthy donors. Moreover, the same group demonstrated that high serum levels of miR-16 and miR-25 correlated with better overall survival than patients with low levels and only an increased miR-25 level was correlated with better progression-free survival [76]. In another study, four miRNAs (miR-1207-5p, miR-3656, miR-630, and miR-483-5p) were up-regulated in MM patients, and eight miRNAs (miR-451, miR-92a, miR-22, miR- 223, miR-19b, miR-720, miR-16, and miR- 20a) were down-regulated in MM patients compared to healthy controls. The plasma levels of miR-483-5p and miR-20a were associated with ISS staging, but only miR-483-5p correlated with progression free-survival, by evidencing its role as a predictive marker of MM survival [77]. In this report, the down-regulation of miR-720 and miR-20a was in contrast with previous studies in which the same miRNAs were found up-regulated [71,73,76]. Down-regulation of miR-19a resulted in a shortened progression-free and overall survival in MM patients and a positive correlation with the ISS stage. Intriguingly, MM patients with low levels of miR-19a had a better response and extended survival after bortezomib treatment. Moreover, the combination of miR-19a and miR-4254 allowed the distinction of MM patients from healthy controls with high sensitivity and specificity [78]. The expression of serum miRNAs was also evaluated in MM and MGUS patients at the moment of diagnosis and at complete response after autologous stem-cell transplantation and in healthy controls. Five serum miRNAs (miR-16, miR-17, miR-19b, miR-20a, and miR-660) were found down-regulated at the diagnosis and increased at the time of complete response. Two miRNAs (miR-19b and miR-331) correlated with longer progression free-survival after autologous stem-cell transplantation, while patients with lower levels of miR-19b or miR-331 had shorter progression free-survival than those with higher levels of either miRNA [79]. Circulating miRNAs were also used to differentiate MM patients with or without extramedullary disease from healthy donors. Circulating miR-130a was found down-regulated in patients with extramedullary myeloma as compared with newly diagnosed, relapsed, and progressed MM patients. Additionally, serum miR-130a discriminated MM patients with extramedullary disease from healthy donors and from newly diagnosed MM patients [80]. Circulating miR-214 and miR-135b were found significantly up-regulated in the serum of patients with bone disease and positively correlated with the severity of the lytic bone disruption. Moreover, patients with high levels of miR-214 had worst progression free and overall survival [81]. Another study reported on the predictive value of circulating miRNAs in patients with relapsed/refractory MM treated with lenalidomide plus low-dose dexamethasone. Of 34 miRNAs differentially expressed between two groups, five miRNAs (miR-26a-5p, miR-29c-3p, miR-30b-5p, miR-30c-5p, miR-331-3p) were significantly lower in poor responders, of which miR-29c-3p showed the highest significance level while the down-regulation of miR-193a-5p was not significant [82]. A summary of the expression profiles for the aforementioned circulating miRNAs in MM is summarized in Table 2.

## 5. Circulating Exosomal miRNAs

Exosomes are lipid-membrane vesicles (30 to 150 nm in diameter) formed through endocytosis and released into the extracellular milieu when the multivesicular bodies fuse with the plasma membrane [83]. The secretion of exosomes, by normal (including B, T, dendritic, mast, and epithelial cells) or tumor cells, can be spontaneous or induced, and can occur under physio and/or pathological conditions [84]. It is believed that their secretion is a fine-tuned process to deliver information to other cells. Exosome uptake/internalization by recipient cells is carried out through direct fusion with the cell membrane or through endocytosis, including clathrin-, caveolin-, lipid-raft-mediated endocytosis, macropinocytosis, and phagocytosis [85,86]. Probably, the uptake depends on the type of cells and the physiologic state, and whether ligands on the surface of the vesicles recognize surface molecules of the cell or vice versa [84,85,87]. Although the mechanisms of biogenesis and sorting are not well defined, several molecules have been shown to regulate these processes, including tetraspanins (D9 and CD63), Rab GTPases (RAB11, RAB35, and RAB27), syndecan-syntenin-ALIX, the endosomal sorting complexes required for transport (ESCRTs), and tumor-susceptibility gene 101 (TSG101) [88]. Exosomes mediate both local and systemic cell–cell crosstalk through transferring proteins, mRNAs, or microRNAs to various cell types, thus altering cell behavior and modifying the microenvironment or altering gene expression in the recipient cells [89]. Moreover, they are also involved with several other mechanisms, such as cell migration and proliferation, tumor survival and invasion, angiogenesis, immune response, and antigen presentation [90,91,92]. In particular, it has been reported that the release of exosomes increases during cancer development [93]. Tumor cell-derived exosomes may carry specific miRNAs that could be used as diagnostic/prognostic tools in myeloma. Importantly, exosomal miRNAs are physically protected from enzymatic degradation, thus increasing the sample integrity for further downstream analysis [94]. Proteomic analysis showed higher expression of oncogenic proteins and cytokines in MM-derived exosomes compared with normal BM stromal cells-exosomes, thus suggesting an exosome-driven interplay between the BM milieu and tumor cells [95,96]. The functional role of exosomes in supporting MM pathogenesis has been investigated. Intriguingly, MM BM-mesenchymal stromal cells secrete exosomes, resulting in induction of tumor growth and MM cell dissemination to the BM niche in vivo. Interestingly, exosomal miR-15a was significantly increased in normal compared to MM BM-mesenchymal stromal cell-derived exosomes, and once released from BM, stromal cells were able to control PC proliferation during MM progression [96]. Another study reported that hypoxic resistant-MM cells secrete a bigger amount of exosomes with respect to the parental cells under normoxia or acute hypoxia conditions. Those exosomes were capable of enhancing new vessel formation in both normoxic and hypoxic human endothelial cells (HUVECS). Of note, exosomal-miR135b, which was found significantly up-regulated in exosomes from hypoxic resistant-MM cells, mediated this mechanism via targeting of the HIF-1*α*-FIH-1 pathway [97]. The same research group investigated the therapeutic potential of healthy-BM stromal cell exosomes derived from donors of different ages using a heterotransplant mouse model of chronic hypoxia-resistant MM cells. A different exosomal miRNAs expression profile characterized young versus older BM stromal cells, and miR-340 was mainly expressed in exosomes derived from young BM stromal cells. In particular, these exosomes significantly inhibited MM-induced angiogenesis, via targeting of the HGF/c-MET signaling pathway in endothelial cells. Interestingly, the transfection of miR-340 into exosomes derived from older BM stromal cells restored the anti-angiogenic properties, thus suggesting that exosomal miRNA replacement could have a therapeutic potential [98]. Recently, circulating exosome-associated miRNAs were also used as prognostic markers to predict primary or acquired drug resistance, by comparing bortezomib-resistant and responsive MM patients. Indeed, four exosomal miRNAs were found down-regulated (miR-16-5p, miR-15a-5p, miR-20a-5p, and miR-17-5p) in the bortezomib-resistant patients with respect to the bortezomib-response patients, by discriminating between the two groups [99]. As previously mentioned, miRNAs are actively involved in MM-bone disease and bone metabolism. Sun et al. recently reported that exosomal miR-214 secreted by osteoclasts is transferred into osteoblasts, via EphrinA2/EphA2 molecules, by inhibiting their activity. Additionally, circulating miR-214 levels were found to be significantly increased in exosomes and serum from osteoporotic patients with respect to the non-osteoporotic ones, thus suggesting its potential function as a biomarker for MM-related bone disease [100]. Recent studies have explored the relationship between exosomal-derived circulating miRNAs levels and patient outcomes, highlighting the prognostic relevance of these miRNAs. Circulating exosomal-miRNAs were isolated from the serum of a large cohort (156 patients) of newly diagnosed MM patients uniformly treated (bortezomib and dexamethasone, followed by high dose melphalan and autologous hematopoietic stem-cell transplant). By performing qRT-PCR array of 22 biologically relevant miRNAs in MM, two miRNAs-derived exosomes, let-7b and miR18a, were found to be significantly correlated with poor patient outcomes with regard to progression-free and overall survival. Importantly, let-7b and miR-18a were predictive in an independent manner even after adjusting for the ISS and specific cytogenetic abnormalities in multivariate and univariate analyses [101].

## 6. Conclusions

Circulating miRNAs may represent novel and promising tumor biomarkers and their potential relevance has been confirmed within the specific context of MM. They are measurable through minimally invasive procedures, such as peripheral blood sampling. Circulating miRNAs have been shown to be valuable prognostic biomarkers as demonstrated by their ability to improve prediction of progression-free and overall survival in patients with MM. Further studies that take into consideration larger cohorts of patients, several disease stages, and different therapeutic settings are required to further corroborate the relevance of circulating miRNAs in MM.

## Figures and Tables

**Table 1:** Differentially expressed miRNAs in MM or MGUS.

miRNAs	Expression	Target	References
miR15a/-16-1	Downregulated in MM	*AKT3, MAPK, rp-S6, NF-kB, VEGF*	[40,41]
miR29b	Downregulated in MM	*MCL-1, Sp1, CDK6, HDAC4, DNMT-3A/-3B, NAFTc, MPP2,* *c-FOS*	[42,43,44,45,46,47,48]
miR-125b-5p	Downregulated in MM	*IRF4*	[49]
miR-23b	Downreguated in MM	*Sp1*	[50]
miR-21	Upregulated in MGUS and MM	*RANK-L, OPG, PTEN, Rho-B, BTG2*	[39,51,52,53]
miR-17-92 cluster	Upregulated in MM	*BIM, SOCS1, SOCS3, SOCS5*	[39,55,56,57]
miR-106-25 cluster	Upregulated in MGUS and MM	*P38 MAPK*	[39,54]
miR-125a-5p	Upregulated in MM	*P53*	[58]

**Table 2:** Circulating miRNAs in different stages of MM.

Upregulated Circulating miRNAs	Disease Stage	Reference
miR-720	MM	[71]
miR-148a miR-221	MM	[71,73,76]
miR-181a miR-625		
miR-20a miR-99b		
miR-142-5p	MM	[74]
miR-660		
miR-29a		
miR-34a	MGUS, MM	[75]
miR-720	MM	[76]
miR-16		
miR-25		
miR-483-5p	MM	[77]
miR-214	MM-Bone Disease	[81]
miR-135b		
**Downregulated circulating miRNAs**		
miR-1308	MGUS, MM	[71]
(5′-cleaved fragment of a GlyGCC tRNA)		
miR-92a	MM	[72,76,77]
miR-744 let-7d	MM	[75]
miR-130a let-7e		
miR-30a	MM	[76,77]
miR-451		
miR-22	MM	[77]
miR-223		
miR-16		
miR-19b		
miR-19a	MM	[78]
miR-17	MM	[79]
miR-660		
miR-130	Extramedullary Myeloma	[80]
